# Effect of Continuous Positive Airway Pressure or Positional Therapy Compared to Control for Treatment of Obstructive Sleep Apnea on the Development of Gestational Diabetes Mellitus in Pregnancy: Protocol for Feasibility Randomized Controlled Trial

**DOI:** 10.2196/51434

**Published:** 2025-04-11

**Authors:** Frances Clements, Hima Vedam, Yewon Chung, John Smoleniec, Colin Sullivan, Renuka Shanmugalingam, Annemarie Hennessy, Angela Makris

**Affiliations:** 1 School of Medicine Western Sydney University Campbelltown, NSW Australia; 2 Department of Respiratory and Sleep Medicine Liverpool Hospital South Western Sydney Local Health District Sydney Australia; 3 Women's Health Initiative Translational Unit (WHITU) Ingham Institute for Medical Research South Western Sydney Local Health District Liverpool Australia; 4 South Western Sydney School of Medicine University of New South Wales Kensington, NSW Australia; 5 Department of Obstetrics and Gynecology Liverpool Hospital Liverpool Australia; 6 Department of Medicine University of Sydney Sydney Australia; 7 Department of Obstetrics and Gynecology Campbelltown Hospital Campbelltown, NSW Australia; 8 Vascular Immunology Research Laboratory The Heart Research Institute University of Sydney Newtown Australia

**Keywords:** obstructive sleep apnoea, OSA, sleep disordered breathing, pregnancy, CPAP, positional therapy, gestational diabetes, GDM, preeclampsia, fetomaternal, maternal, pregnant, fetus, fetal, breathing, apnoea, sleep, respiratory, eclampsia, pregnant women, pregnancy complications, hypertension

## Abstract

**Background:**

Obstructive sleep apnea (OSA) is a common sleep disorder, and in pregnancy, it is associated with an increased risk of complications, including gestational diabetes mellitus and preeclampsia. Supine sleep may worsen OSA, and in pregnancy, it is associated with an increased risk of stillbirth due to effects on fetomaternal blood flow. Continuous positive airway pressure (CPAP) therapy is considered the gold-standard treatment for moderate to severe OSA, although compliance is frequently poor; positional therapy (PT) is generally less effective than CPAP in nonpregnant patients but may be better tolerated and more accessible during pregnancy. There is limited data on whether widespread, early screening for sleep disorders in pregnant women with symptoms of sleep-disordered breathing or at high risk of metabolic complications and subsequent early intervention with CPAP or PT attenuates fetomaternal risks.

**Objective:**

This study aims to determine the feasibility of conducting a randomized controlled trial to assess improved fetomaternal outcomes in a high-risk pregnant population with OSA, using CPAP or PT, initiated by the 16th week of gestation.

**Methods:**

This study is a randomized, controlled, open-label feasibility study in which pregnant women with an apnea-hypopnea index (AHI) or respiratory disturbance index (RDI) ≥5 are treated with CPAP (auto-titrating and fixed pressure) or positional therapy from early gestation (by 16 weeks) until delivery. The primary outcome is the feasibility of the study protocol and the development of gestational diabetes mellitus by the 28-week gestation period. Secondary outcomes include the development of hypertensive disorders of pregnancy (HDP), maternal weight gain, uterine artery blood flow, glycemic control during pregnancy (in participants who develop gestational diabetes), changes in maternal circulating biomarkers, and neonatal birthweight complications. Polysomnography at 28- to 32-week gestation period, postpartum polysomnography, therapy compliance, and patient acceptability are also assessed.

**Results:**

The trial commenced on September 30, 2019. The trial is ongoing as of August 6, 2024.

**Conclusions:**

The trial intends to contribute to the growing evidence base to support the need for the identification and treatment of OSA occurring during pregnancy and to assess the feasibility of the study protocol. This will be the first trial to compare the early initiation of CPAP (auto-titrating and fixed pressure) and positional therapy in pregnant women from early gestation, providing alternative therapies for the treatment of OSA in this important population.

**Trial Registration:**

Australian New Zealand Clinical Trials Registry ACTRN12619001530112; https://tinyurl.com/yctdzs4u

**International Registered Report Identifier (IRRID):**

DERR1-10.2196/51434

## Introduction

During pregnancy, physiological changes, including upper airway edema and hormone-related upper airway pressure changes, predispose women to sleep-disordered breathing (SDB) [1]. The prevalence of obstructive sleep apnea (OSA) in pregnancy increases from 3.6% in early pregnancy to 8.3% in midpregnancy and is associated with an independent risk of gestational diabetes mellitus (GDM), preeclampsia, and hypertensive disorders of pregnancy (HDP) [2]. In addition, emerging evidence suggests that the presence of SDB before pregnancy may place women at an increased risk of developing GDM [3]. Supine sleeping in late pregnancy is associated with an increased risk of stillbirth [4], and supine sleep avoidance is recommended from the 28-week gestation period [5].

Treatment options for OSA in non-pregnant adults include continuous positive airway pressure (CPAP), weight loss, positional therapy, mandibular advancement splints, surgery (both upper airway and bariatric), and pharmacological therapies, though not all are suitable for use in pregnant populations. CPAP and positional therapies reduce the occurrence of OSA by reducing the occlusion of the upper airway through pneumatic splinting [6] and repositioning the sleeping persons to reduce collapsibility of the airway [7,8], respectively. Within nonpregnant populations, longer duration CPAP compliance (more than 7 hours) was associated with a reduction in hemoglobin A_1c_ by 1% when CPAP was used for more than 85% of the rapid eye movement stage of sleep during the night [9]. Improvements in blood pressure control have been demonstrated in nonpregnant patients with OSA [10,11], and a small benefit in 24-hour diastolic blood pressure in patients using fixed pressure CPAP compared with auto-titrating CPAP has been reported [12].

In pregnancy, CPAP is recommended as the first line of treatment for OSA [13,14], although alternatives to CPAP may be beneficial but require clinical trials to investigate their utility [14]. In pregnant populations, many studies investigating CPAP treatment have used small sample sizes [15] or initiated therapy in later gestation periods. An early study of 12 pregnant women with OSA at risk of preeclampsia demonstrated no improvement in clinical maternal outcomes [16]; likewise, CPAP failed to demonstrate improvements in glucose levels in obese pregnant women with 2 weeks of CPAP use between 24- and 34-week gestation periods, although compliant use of CPAP in this study demonstrated an improvement in insulin secretion and sensitivity [17]. There are, however, some benefits reported in the literature; a single night of CPAP in a study of 10 pregnant women with preeclampsia and coexisting obesity demonstrated improvements in fetal movements [18], and an improvement in blood pressure control both during sleep and wake was reported in a small study of 7 women, resulting in down titration of antihypertensive medication [19].

Compliant use of CPAP remains a challenge, and in some patients, it may be dependent on clinical support mechanisms, with pre- and postacclimatization phase support improving therapy compliance [20]. Gender-based differences in CPAP compliance have been demonstrated in a large study of nearly 800,000 CPAP users, where women aged 18-30 had the lowest CPAP compliance (51.3%) and were most likely to cease therapy [21]. Barriers to compliant CPAP therapy in pregnant women with OSA are relatively unknown though targeted supports, which have demonstrated improvements in maternal medication compliance, may be beneficial [22]. A recent randomized controlled trial assessed blood pressure outcomes in 340 high-risk pregnant women recruited predominantly during the first trimester, allocated to CPAP or no treatment (control). Despite a mean CPAP compliance of 2.5 hours nightly, and an overall compliance (≥ 4 hours nightly) of 32.7% of participants randomized to CPAP, this reported reductions in diastolic blood pressure and preeclampsia rates [23]. This study additionally presents data on CPAP side effects, and despite rhinitis, mask difficulties, and pressure intolerance reported, there was no significant difference in the reporting of these symptoms between compliant and noncompliant participants, indicating the possibility that these side effects are not barriers to treatment in this special population.

CPAP devices are available in fixed and auto-titrating options and in nonpregnant OSA patients, patient preference has demonstrated mixed results [24,25], although data are lacking in pregnant populations. Furthermore, evidence of the efficacy of auto-titrating devices through gestation advancement is limited, and despite the availability of auto-titrating devices that attempt to target flow-limited breathing using reduced air pressure in premenopausal women [26], their utility in pregnancy is unknown.

Positional therapy is less effective at reducing apnea-hypopnea index (AHI) and nocturnal oxygen desaturation [7,27] but is effective at reducing supine sleep time [7], which is associated with the worsening of OSA [8]. Positional therapy (PT) in pregnancy is hypothesized to impact outcomes by two potentially independent pathways: first, by addressing SDB, which is typically worse in the supine position, and second, by minimizing compression of the vena cava by the gravid uterus, which may reduce fatal blood flow. A feasibility study using a back-worn positional therapy band for a single night in healthy pregnant women at 32- to 38-week gestation periods showed improvement in maternal oxygenation and reduced fetal heart rate decelerations compared with the controlled night [28].

Given placentation completes around 16-week gestation period, the timing of OSA treatment intervention may be particularly important, as data demonstrates significant benefits of time-specific interventions in pregnancy, for example, aspirin is generally beneficial in preventing preeclampsia if commenced before 16-week gestation period [29]. We thus outline a protocol to determine the feasibility of a parallel group, individually randomized, controlled, open-label trial comparing CPAP or PT to usual care (control). Commencement of therapy by the 16th week of gestation has been chosen to reduce exposure to intermittent hypoxia on the developing placental function during early pregnancy and to establish early compliance to treatment. The use of fixed pressure CPAP after auto-titration of pressure in this trial was determined by factors including access to trial equipment, cost, and absence of evidence of the superiority of auto-titrating over fixed pressure CPAP in pregnancy. This trial aims to determine the feasibility of conducting a larger trial and to obtain preliminary recruitment and efficacy data that may be used to design and power a larger trial. This study may be of interest to others conducting sleep therapy intervention studies in pregnant women.

### Clinical Trial Objectives

This study aimed to determine whether CPAP or PT initiated by the 16th week of gestation in pregnant women with a respiratory disturbance index (RDI) or AHI ≥5 results in improved clinical outcomes, particularly in relation to the development of gestational diabetes.

### Trial Feasibility Objectives

This study aims to assess the feasibility and acceptability of trial interventions and schedule of visits and determine limitations of trial design, including patient and hospital staff participation.

## Methods

All trial primary and secondary outcomes are described in the Australian New Zealand Clinical Trials Registry Trial Registry.

### Ethics Approval and Consent to Participate

#### Overview

Participant recruitment commenced on September 10, 2019. The final patient was recruited in December 2022 and is expected to complete participation in June 2024. The participants affected by product recalls in this trial will be followed up annually for 5 years, if agreeable as per the protocol. Data from participants who choose to withdraw from study participation will be included in the final analysis unless requested by the participant. The trial was registered on November 6, 2019 (ACTRN12619001530112), 7 weeks after the first participant was recruited for the study. The study protocol was not amended during this time.

#### Human Subject Ethics Review Approvals or Exemptions

The South Western Sydney Local Health District Human Research and Ethics Committee (SWSLHD HREC) approved this study (June 12, 2019, project identifier 2019/ETH00283). The current protocol version 3.1 was approved on November 11, 2022.

#### Informed Consent

The participants with scheduled antenatal bookings at Liverpool and Campbelltown hospitals will be invited to undertake a screening eligibility preconsent questionnaire, which is presented in Multimedia Appendix 1 by research study staff to determine participation eligibility as described in the eligibility criteria above. Women will be sequentially approached.

Following the screening, study staff will invite eligible participants to provide written informed consent. Multimedia Appendix 2 presents the postconsent baseline information questionnaire, which will be collected at the next trial consent.

The participants will undertake attended polysomnography to determine eligibility in the RCT. Additionally, the participants will be invited to undertake Apnealink Air and Somte at both the baseline and 28- to 32-week gestation periods, in addition to the attended polysomnography as part of a substudy assessing agreement in AHI or RDI scores in the early to mid-gestation period. Sleep studies will be undertaken within a 7-day window, where practicable. Completion of Apnealink and Somte are not required for RCT participation but will be used to determine the validity of these tests for future use in this population. Eligibility for participation in RCT will be determined by polysomnography outcome.

Women who undergo polysomnography by 14-week gestation period and demonstrate OSA as evidenced by an RDI or AHI ≥5 on polysomnography will be invited to participate in the RCT provided exclusion criteria are not met as described above. A second written informed consent will be collected by study staff, and the participants will be randomized to CPAP, PT, or control as described in the randomization section below.

Validation of home sleep testing in this population will be undertaken via a separate substudy. After validation of the home testing method, further recruitment and screening measurements will continue with screening questionnaires and home Apnealink Air only.

#### Privacy and Confidentiality

Unattended polysomnography, Apnealink Air, and biomarker data will be deidentified, as described in the relevant methods sections below. Patient data are deidentified for the purpose of randomization, as described below.

Participant consent forms, printed results, and other identifying information on hard copy will be stored in a locked cabinet in a security-access area of the Respiratory and Sleep Medicine Research Department at Liverpool or Campbelltown Hospitals. Where possible, study data will be stored separately to participant identifiers. A master list of study ID numbers and participant identifiers will be stored electronically in a password-protected file within a designated electronic folder on the Respiratory Research drive on the secure Liverpool Hospital server. A separate password-protected folder will be designated for any electronic records with results and identifying information.

#### Compensation

If the participants suffer any harm, they will be compensated as per health authority regulations.

### Trial Design

Pregnancy-associated obstructive sleep apnea (POSA) is a 2-center randomized controlled feasibility trial. The participants will be recruited following participation in an observational study by our group that assesses diagnostic validation of Apnealink air and Somte devices and long-term fetomaternal outcomes. The participants will also be approached at antenatal visits or called by telephone to assess eligibility. Recruitment at both study sites will be concurrent, and the participants who meet the eligibility criteria based on AHI or RDI results in the observational study will be approached for participation in the RCT.

The RCT will use a parallel group, individually randomized, open-label design with two interventions and one control group (1:1:1), with minimization allocation among the 3 arms stratified for BMI, previous history of preeclampsia, or gestational diabetes mellitus. A total of 48 participants (16 in each arm) will be recruited. Study visits will occur monthly after randomization until 32-week gestation period and fortnightly thereafter until delivery, as is local routine clinical practice for antenatal visits. Data collection will include demographic, clinical and physical details, sleep questionnaire responses, sleep study data, maternal pathology, fetal scans, birth details, maternal and fetal clinical outcomes, sleep therapy compliance, and therapy acceptance questionnaires.

We used the SPIRIT (Standard Protocol Items: Recommendations for Interventional Trials) checklist when writing our protocol [30], which can be found in Multimedia Appendix 3.

### Trial Setting

POSA will be conducted at Liverpool Hospital (LPH) and Campbelltown Hospital (CTN) public hospitals within the South Western Sydney Local Health District in metropolitan Sydney, New South Wales, Australia. LPH is a tertiary referral center offering comprehensive antenatal services, delivering approximately 4000 women annually, and includes a level 6 neonatal intensive care unit for neonates from a 24-week gestation period in the district and a comprehensive sleep and respiratory service, including a 5-bed sleep laboratory, ambulatory testing, and a comprehensive outpatient service, including clinics for CPAP therapy. CTN delivers approximately 4200 women annually, with a level 4 nursery that cares for neonates from a 32-week gestation period and a general respiratory and sleep clinic, without the capacity to undertake polysomnography. POSA participants recruited from CTN will be referred to LPH for laboratory polysomnography.

### Eligibility Criteria

Eligible participants are defined as women aged 18 years of age and older; in early pregnancy (up to 14-week gestation period); at increased risk of metabolic complications defined as one or more of (1) BMI greater than or equal to 35 kg/m^2^, (2) previous GDM, (3) previous personal history of preeclampsia (or in mother or sister), (4) underlying renal disease, (5) maternal type 2 diabetes (pregestational), and (6) symptoms of SDB including snoring, witnessed apnea, mild excessive daytime sleepiness (EDS; which does not meet the criteria for severe EDS defined by Epworth Sleepiness Scale (ESS>15), or a fall asleep accident, or near-miss accident in the previous 12 months), or tiredness; and obstructive sleep apnea diagnosed by polysomnography defined as RDI or AHI ≥ 5.

The participants will be excluded if they have (1) a previous diagnosis of OSA on active treatment, (2) confirmed GDM or preeclampsia, (3) maternal type 1 diabetes, (4) multifetal gestation, (5) known fetal chromosomal abnormality, (6) inability to provide informed consent, and (7) severe EDS based on clinical assessment (eg, including a fall asleep motor vehicle accident or near miss, transient sleepiness while driving or at lights or needing to pull over due to sleepiness while driving, or transient sleepiness in any other dangerous situation, ie, cooking, carrying a baby) or ESS of greater than 15.

### Interventions

All groups will have antenatal care as usual without restriction during the trial. The participants will be randomized to CPAP, PT, or control, and the participants randomized to intervention (CPAP or PT) will undertake therapy device education at the randomization visit and commence therapy on the day of randomization or as soon as is practicable. The participants will also be encouraged to use their therapy device for all sleep periods (including naps) from randomization until delivery. CPAP and PT devices will be returned following delivery or withdrawal or discontinuation from the study. The CPAP masks used by the study participants may be retained at the end of the study.

All participants will undertake polysomnography at the 28- to 32-week gestation period. CPAP or PT intervention participants will complete polysomnography at a 28- to 32-week gestation period with the device in situ to confirm treatment efficacy. CPAP will be titrated, if required, as described above. In addition, participants will be invited to complete Somte and Apnealink at 28- to 32-week gestation period, as part of the device validation substudies.

### CPAP Intervention

The participants in the CPAP intervention group will use a close-fitting facial mask worn overnight during sleep to ensure that the collapsible upper airway is kept patent with a column of air and they will commence on auto-titrating pressure 6-20 cm H_2_O (Philips Dreamstation Auto with heated humidification) per clinical practice at the study site. The participants will also be offered a range of CPAP masks to trial with an option to take home multiple masks or sizes if needed. CPAP participants will be educated in device use and troubleshooting by an experienced sleep therapist before commencing therapy. The trial sleep therapist will telephone CPAP group participants on day 3 to gain access to CPAP efficacy data via remote monitoring software (Care Orchestrator, Philips). Pressure will be changed to the 90th percentile pressure in fixed pressure mode, provided compliance data show average usage of CPAP across a period of more than 4 hours (All Days≥4 hours), and treatment efficacy is achieved as determined by residual AHI≤5. The participants with poor compliance or efficacy will be given additional acclimatization time on CPAP, up to 7 days (or as clinically necessary), and changed to the 90th percentile when clinically appropriate. CPAP data will be downloaded via Secure Digital card (SD) data card and reviewed at each study visit and at the return of the device as per the study schedule and interrogated for CPAP compliance, efficacy, and mask leak.

CPAP pressure will be adjusted empirically at scheduled study visits to maintain treatment efficacy (AHI≤5). Polysomnography will be conducted at 28 weeks on the current CPAP pressure. Titration of CPAP pressure will be undertaken if criteria for pressure increase is met per AASM (American Academy of Sleep Medicine) manual titration guidelines, that is, up to 1 increase by 1 cm H_2_O per 5-minute period for any of the following: 2 of obstructive apneas, 3 of hypopneas, 5 of the respiratory event–related arousals, or 3 minutes of loud or unambiguous snoring [31]. CPAP pressure changes will be reported in published study findings at the conclusion of the trial.

Compliance and efficacy of treatment feedback will be provided to study participants at each visit to encourage compliance. Additional phone or video support will be available, at participants’ request, for participants who encounter difficulty with CPAP compliance. To ensure equal therapy support for CPAP and PT treatment arms, remote monitoring of CPAP compliance will not be conducted beyond the acclimatization period. CPAP devices used in this trial have been replaced by the manufacturer in accordance with the Therapeutics Goods Administration device repair or replacement program following product defect correction order RC-2021-RN-01373-1.

### Positional Therapy Intervention

**Figure 1:**
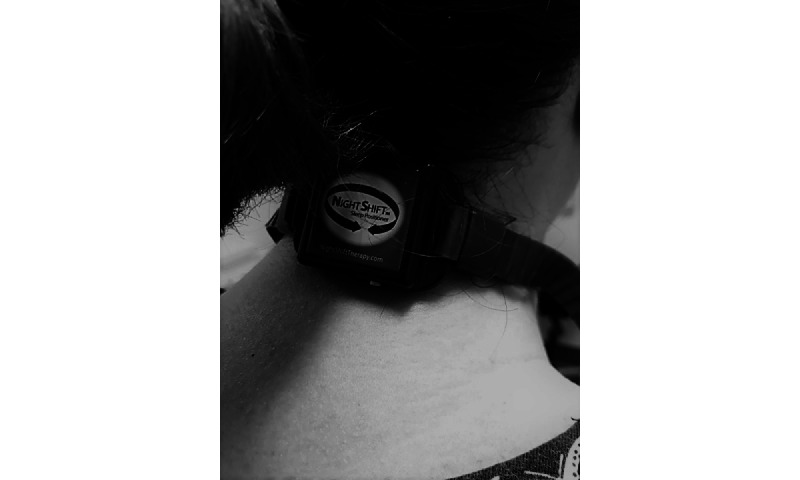
Nightshift positional therapy device posterior view.

**Figure 2:**
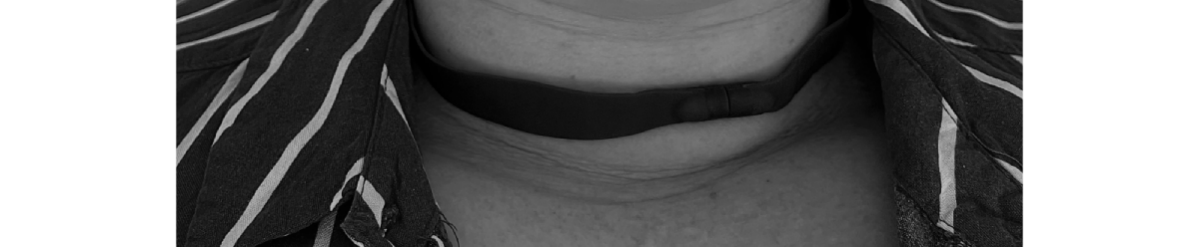
Nightshift positional therapy anterior view.

The participants in the PT intervention group will use a noninvasive vibratory device (Night Shift Device: Advanced Brain Monitoring, Inc) that is worn around the neck and promotes sleep in the nonsupine position (Figure 1 [source: Frances Clements, Liverpool Hospital, November 10, 2022])*.* The device provides a vibratory alert to the wearer when the supine position is detected and is secured at the anterior neck with a magnetic closure (Figure 2 [source: Frances Clements, Liverpool Hospital November 10, 2022]).

The participants randomized to PT will be provided education by an experienced sleep therapist before commencing therapy. The device will be fitted by measuring the neck circumference and adjusted to ensure that the device does not slide down the neck. The device will be set with a 15-minute feedback delay, with vibration feedback mode on and the device location at the posterior neck. Downloads of therapy data will be obtained by an experienced sleep therapist at each study visit, where possible, as per the protocol schedule, and at the return of the device, compliance and efficacy data will be interrogated. Recording time, position, patient alert frequency, and snore data will be recorded. Feedback will be provided to study participants at each visit. Additional phone or video support will be available for participants with therapy as requested.

### Control Group

Control group participants with significant SDB at polysomnography at the 28- to 32-week gestation period and symptoms of severe EDS (ESS>15) will be identified, withdrawn from the study, and managed according to standard clinical practice under the direction of a sleep physician.

### Participant Timeline

The participants will be reviewed monthly until the 32-week gestation period and then fortnightly until delivery, coinciding with scheduled antenatal visits where possible. The trial flow chart is depicted in Multimedia Appendix 4. The flowchart describes the participant's progression through the trial protocol and depicts the timing of the interventions.

### Sample Size

There are limited data on this topic, particularly on the estimated risk reduction in event rates. We intend to pilot the RCT with an initial 48 patients. This data will be used to refine the protocol and optimize logistics and will be used to inform a larger RCT.

### Randomization, Allocation, Concealment, and Blinding

The participants will be individually randomized via a telephone call to the National Health and Medical Research Council’s Clinical Trials Centre at Sydney University, who are independent of the trial, by the study coordinator. Randomization will be based on a minimization method with stratification for previous preeclampsia, personal history of gestational diabetes, and BMI≥35, to either CPAP, PT, or control groups. A maximum imbalance value of 1 between any of the 3 arms will be used in determining whether a forced allocation (ie, to achieve minimization of difference of stratification factor totals) or random allocation is used in a specific subject randomization. Treatment allocation will be unblinded for both study staff and participants.

### Data Collection, Management, and Analysis

The complete schedule of study visits, procedures and data collection points is presented in Multimedia Appendix 5, which depicts the trial participant study visits and intervention schedule.

### Data Management

Data from Apnealink screening tests, CPAP, and PT will be stored in a deidentified format. Polysomnography data will be stored on the secure hospital network. The system is secured via the Information Management and Technology Division’s corporate backup software. Research Electronic Data Capture (REDCap; Vanderbilt University) database [32] will be used for data collection and management. Questionnaires will be completed electronically by participants via an email link where possible or by paper questionnaire where needed. In the event of participant discontinuation from the RCT, long-term fetomaternal outcomes of interest will be collected, where agreed to by participants during consent, in writing.

### Data Collection Methods

#### Blood and Urine Collection

With consent, blood will be collected at 12-16, 16-20, 28 gestation weeks, and 6-12 months post partum by study staff or using hospital pathology collection services in EDTA tubes. The blood will be centrifuged (3000 rpm, 10 min), and plasma will be aliquoted (250 µL). The buffy coat will be collected and stored in approximately 250 µL RNA. Later, all specimens will be snap frozen in liquid nitrogen and stored at –80C at the Ingham Institute of Medical Research (Liverpool, New South Wales, Australia). Biomarkers (sFlt-1 [soluble fms-like tyrosine kinase 1], PlGF [placental growth factor], IL-6 [interleukin-6], TNFα [tumor necrosis factor alpha], and HIF1α [hypoxia-inducible factor 1-alpha]) will be assayed at the conclusion of the study blinded to outcomes.

Urinalysis will be conducted at each study visit using SIEMENS Multistix 10SG Test strips. A score of negative, trace, 30 mg/dL (++), 100 mg/dL (++), 300 mg/dL (++), or >2000 mg/dL (+++) will be recorded. If greater than 1+ protein is detected, a random spot urine sample will be sent to hospital pathology services for measurement of urinary protein adjusted for creatinine excretion.

#### Fetal Monitoring

Fetal ultrasound will be undertaken at the study site fetomaternal units (FMUs). Uterine artery blood flow (pulsatility index measurements) will be recorded at the randomization visit (12-16-week gestation period), 2-6 weeks later (average 4 weeks to 16-20-week gestation period), and at 36-week gestation period. Morphology scan (16 weeks) (may be combined with uterine artery blood flow) and growth scan (36 weeks) will be performed. Fetomaternal scans performed at study sites will be performed using ViewPoint 6 (General Electric Healthcare) by experienced sonographers supervised by physician consultants within the FMU.

#### Questionnaires

ESS [33] and STOP-BANG (snoring, tiredness, observed apnea, high BP [STOP] and snoring, tiredness, observed apnea, high BP-BMI, age, neck circumference, and gender) [34] questionnaires will be used in baseline screening and scored according to standing scoring recommendations (sum of 8 item-scores [ESS] and score 0-8 [STOP BANG]). Functional outcomes of sleep will be assessed using the Functional Outcomes of Sleep Questionnaire (FOSQ-10) [35], which has been validated in pregnancy and scored according to FOSQ-10 marking guidelines [36]. Questionnaires assessing sleep study acceptability, sleep study preference, and therapy acceptability will be undertaken and are presented in Multimedia Appendices 6-13. Questionnaires will be emailed to participants using REDCap or completed in paper hardcopy where the patient is unable to complete electronically. The acceptability questionnaires used in this trial were evaluated on hospital staff unaffiliated with the trial. Feedback was implemented, and questionnaires were redesigned before SWSLHD HREC approval and implementation.

#### Sleep Study Data Collection

The participants will undergo attended polysomnography (with therapy device in situ for CPAP and PT arms) using Grael v2 (Acquisition System, Compumedics), unattended polysomnography using SOMTE v2 (Acquisition system, Compumedics) and Apnealink Air (Resmed) at 28- to 32-week gestation period at LPH. Laboratory-based polysomnography will be repeated 6-12 months post partum.

Attended and unattended polysomnography will be scored using Profusion PSG 4 auto analysis software (Compumedics) by a single experienced sleep scientist using the 2020 AASM Manual for the scoring of sleep and associated events (Version 2.6 guidelines). Apnealink Air will be automatically scored using Airview (Resmed; AASM 2012, Automatic Scoring) and manually scored by a single experienced sleep scientist using 2020 AASM guidelines (events Version 2.6). All sleep study sets will be reported by the same sleep physician for consistency. Unattended polysomnography and Apnealink Air studies will be scored and reported blindly by sleep scientists and reporting sleep physicians.

Apnealink Air data collection includes airflow (pressure transducer), respiratory effort (abdomen) and oximetry (SpO_2_), snoring, and pulse. The participants will complete a self-setup in the home, will be provided with an instruction sheet, and will also manually start study recording as close to anticipated sleep onset as possible and manually stop recording at sleep offset.

Unattended polysomnography data collection included electroencephalography (F3 and F4, M1 and M2), electrooculography (E1 and E2), electromyography (EMG chin [submentalis]), electrocardiogram (modified lead II), airflow (pressure transducer), snoring, airflow (thermistor), respiratory effort (abdomen and thoracic), anterior tibialis EMG (left and right leg), oximeter (SpO_2_), and position sensor. The participants will complete a self-setup at home. A comprehensive instruction booklet will be provided, and the participant will be encouraged to watch an instructional video [37]. The participants will have access to phone or video support from experienced sleep staff. Study recording will commence as close to the anticipated sleep onset as possible and manually stop recording at sleep offset.

Attended polysomnography collection includes those in unattended polysomnography as described above, with the addition of electroencephalography (C1 and C2 and O1 and O2), EMG Chin (Chin 1 and Chin 2 and Chin 3), EMG diaphragm, snore (microphone), sound level (dB meter), and digital video (audio and visual). Competent sleep technicians will conduct participant setup.

### Outcome Assessment

An oral glucose tolerance test will be undertaken for the assessment of gestational diabetes at 28 weeks (in addition to earlier testing if clinically requested). Collection of fasting blood will be followed by administration of 75 g oral glucose load, with 1 and 2 hours of blood collection. Interpretation will follow the Australasian Diabetes in Pregnancy Society (ADIPS) 2020 guidelines [38].

Blood pressure (BP) will be recorded at each study visit. Diagnosis of HDP will be defined as per the Society of Obstetric Medicine of Australia and New Zealand (SOMANZ) guidelines [39].

Anthropometric measurements (height, weight, and BMI) will be recorded during the randomization visit, and weight will be checked at subsequent study visits by study staff using hospital clinic scales.

### Postpartum Follow-Up

RCT participants will be followed up in the clinic at 6-12 months post partum. At this time point, women will be offered repeat polysomnography, an oral glucose tolerance test (if they developed diabetes and have not already undertaken this in the postpartum period), biomarker blood, and urine collection.

### Statistical Methods

This trial is a feasibility trial, and as such, no power calculations for the RCT were derived. We prespecified to assess the diagnostic accuracy of the home sleep tests as well as the screening questionnaires following the STARD (Standards for Reporting of Diagnostic Accuracy) guidelines. The accuracy will be assessed at various cutoff points using a receiver operating characteristic curve. The area under the receiver operating characteristic curve and positive and negative predictive values in the patient population will be derived. The first recruitment target will be 50 participants, which provides at least 80% power (α=.05) to detect a C-statistic of 0.7 when the allocation ratio is 1 (ie, 50% prevalence) and 0.75 when the allocation ratio is 2 (ie, 20% prevalence). We will be comparing Apnealink Air and SOMTE unattended PSG to attended PSG. Further exploratory analysis will be undertaken to assess whether any factors at booking, including the results of the sleep questionnaire, biomarkers, clinical factors, and history in combination with findings of unattended PSG, Apnealink, or attended PSG, are predictive of poor outcomes (either maternal or fetal, as discussed above). A composite of the combined adverse maternal outcomes and combined adverse fetal outcomes will also be assessed as an outcome. Data will be displayed as mean (SD), 95% CIs, and median (IQR) as appropriate based on data distribution. An intention-to-treat analysis will be undertaken for the primary outcome as well as the secondary outcomes. A per-protocol analysis (for primary and secondary outcomes) will also be undertaken, and both data will be presented. A *P* value of <.05 will be considered statistically significant. Where multiple analyses are undertaken, adjustments will be made for multiple comparisons. Longitudinal analysis of repeated measures will be undertaken where measurements are undertaken several times. Where differences at baseline exist between the groups, adjustments will be made statistically. Where less than 10% of any data point is missing, data will be imputed. We have preplanned an exploratory analysis of flow characteristics of all sleep tests to investigate sub-criterion flow changes related to long-term fetomaternal outcomes.

### Patient and Public Involvement

The participants were not involved in the design of this study protocol. Participant feedback will be obtained via a series of questionnaires as described above. Members of the public (hospital staff) participated in providing feedback on acceptability questionnaires as described above, and feedback was implemented in the development of the questionnaires.

### Data Monitoring

No data monitoring committee has been appointed.

### Risk Assessment and Termination of Study

An interim analysis will be performed halfway through enrollment by the safety committee. If there is a convincing signal that either CPAP or PT therapy is associated with adverse outcomes, the study will be terminated immediately, and the SWSLHD HREC will be notified. Significant safety issues and patient complaints will be reported immediately via email to SWSLHD HREC.

### Safety Considerations

A safety committee will be established to monitor the safety of participants and to review any potential adverse outcomes. The safety committee will review the outcomes after each of the 4 subjects for the first 16 subjects or if any severe adverse events occur.

### Posttrial Care

The participants affected by product recalls in this trial will be followed up annually for 5 years if agreeable as per the protocol.

## Results

The trial commenced on September 30, 2019. The trial is ongoing as of August 6, 2024.

## Discussion

### Principal Findings

Despite the growing interest in the potential for improved fetomaternal outcomes in pregnant women with OSA occurring during pregnancy through the use of CPAP, few large RCTs have been undertaken. In addition, no RCT has compared CPAP and positional therapy for the treatment of OSA occurring during pregnancy. This RCT feasibility study will assess the feasibility of conducting a large RCT to initiate CPAP or positional therapy in pregnant women by the 16th week of gestation.

To date, the largest RCT to investigate the treatment of OSA in pregnant women studied 310 pregnant women with OSA from the first trimester. The primary outcome of the study was BP control during pregnancy and the secondary outcome was the incidence of preeclampsia. The overall CPAP compliance was 2.5 (SD 2.5) hours per night and median use was 1.7 (IQR 0.2-4.5) hours per night, and the authors concluded CPAP therapy reduced the incidence of preeclampsia and demonstrated a reduction in DBP throughout the pregnancy, in high-risk pregnant women with mild to moderate OSA [23]. A recent systematic review assessed the use of CPAP therapy for the prevention of hypertensive-related adverse outcomes in pregnant women with OSA. The authors report risk reduction of gestational hypertension and preeclampsia in the CPAP groups (relative risk [RR] 0.65, 95% CI 0.47-0.89; *P*=.008) and preeclampsia (RR 0.70, 95% CI 0.50-0.98; *P*=.04), which were not correlated with age or BMI. The authors concluded that while the results suggest CPAP therapy as a potential mediator to adverse gestational hypertensive outcomes in pregnant women with OSA, the association remains inconclusive [40].

This feasibility RCT will assist in the understanding of the maternal and health care system challenges in undertaking a CPAP intervention during pregnancy. The therapies in this study will commence by the 16th week of gestation and will be overseen by an experienced sleep therapist to improve CPAP and PT compliance. This is important as many clinical trials involving CPAP are limited by poor compliance of the participants. We will encourage compliance to interventions through an initial education session at randomization by the trial sleep therapist and at each study visit. At these visits, the participants will be provided feedback regarding their therapy compliance and efficacy, and through a series of questionnaires, we will assess barriers to therapy. We anticipate an improvement in fetomaternal outcomes such as improved blood pressure control, reduced incidence of GDM, and preeclampsia in the interventional groups with good adherence to therapy. The learnings from undertaking this work will be important for consideration when expanding to a larger trial. The results of the study will be published in peer-reviewed journals and presented at national and international conferences.

### Limitations

Limitations to the trial will be poor acceptability of the trial protocol by the participants and the potential for pregnancy complications to participants in the trial, given the risk of adverse events occurring during pregnancy, particularly in high-risk populations with comorbidities such as obesity and a history of hypertension and diabetes [41]. Additional limitations to the trial will be the potential for poor CPAP and PT compliance of the participants randomized to these interventions. The use of an experienced sleep therapist in the trial to educate the participants and provide ongoing therapy support during the trial is anticipated to minimize poor therapy compliance but may limit the generalizability of findings.

### Conclusions

If this trial protocol demonstrates good overall feasibility, a larger multicenter RCT will be conducted. The results of this feasibility trial will inform a redesign of the protocol for the larger RCT, if required. The use of positional therapy in this trial may provide evidence of the utility of OSA treatment options other than CPAP in pregnant women with OSA. Furthermore, it is anticipated that the follow-up schedule in this trial may inform other study designs involving pregnant women with OSA, ultimately leading to changes to clinical policy in the care of pregnant women.

## Appendix

Multimedia Appendix 1 Screening eligibility preconsent questionnaire. Participants are invited to complete a Screening eligibility preconsent questionnaire to determine trial eligibility.

Multimedia Appendix 2 Baseline information. Participants complete the baseline information questionnaire following trial consent.

Multimedia Appendix 3 The checklist presents the Standard Protocol Items: Recommendations for Interventional Trials (SPIRIT) 2013 checklist.

Multimedia Appendix 4 POSA (pregnancy-associated obstructive sleep apnea) trial flow chart. The flow chart describes the participant progression through the trial protocol and depicts the timing of the interventions.

Multimedia Appendix 5 Schedule of study visits. The image depicts the trial participant study visits and intervention schedule.

Multimedia Appendix 6 Polysomnography (PSG) 28-32 weeks gestation questionnaire. Participants are invited to complete the questionnaire at 28-32 weeks' gestation to assess participant experience following completion of the sleep test.

Multimedia Appendix 7 Somte 28-32 weeks gestation questionnaire. Participants are invited to complete the questionnaire at 28-32 weeks' gestation to assess participant experience following completion of the sleep test.

Multimedia Appendix 8 Apnealink 28-32 weeks gestation questionnaire. Participants are invited to complete the questionnaire at 28-32 weeks' gestation to assess participant experience following completion of the sleep test.

Multimedia Appendix 9 Preferred test questionnaire. Participants are invited to complete the questionnaire to assess participant preference of sleep diagnostic tests.

Multimedia Appendix 10 CPAP (continuous positive airway pressure) therapy Questionnaire 16-20 weeks' gestation. Participants randomised to CPAP intervention are invited to complete the questionnaire to assess participant experience of CPAP therapy to 16-20 weeks' gestation.

Multimedia Appendix 11 CPAP (continuous positive airway pressure) therapy questionnaire 36 weeks' gestation. Participants randomised to CPAP intervention are invited to complete the questionnaire to assess participant experience of CPAP therapy to 36 weeks' gestation.

Multimedia Appendix 12 Position therapy questionnaire 36 weeks' gestation. Participants randomised to positional therapy (PT) intervention are invited to complete the questionnaire to assess participant experience of PT therapy to 36 weeks' gestation.

Multimedia Appendix 13 Position therapy questionnaire 16-20 weeks' gestation. Participants randomised to positional therapy (PT) intervention are invited to complete the questionnaire to assess participant experience of PT therapy by 16-20 weeks' gestation.

## Abbreviations

AASM: American Academy of Sleep Medicine
ADIPS: Australasian Diabetes in Pregnancy Society
AHI: apnea-hypopnea index
BP: blood pressure
CPAP: continuous positive airway pressure
CTN: Campbelltown Hospital
EMG: electromyography
ESS: Epworth Sleepiness Scale
FMU: fetomaternal unit
FOSQ-10: Functional Outcomes of Sleep Questionnaire
GDM: gestational diabetes mellitus
HDP: hypertensive disorders of pregnancy
HDP: hypertensive disorders of pregnancy
HIF1α: hypoxia-inducible factor 1-alpha
IL-6: interleukin-6
LPH: Liverpool Hospital
OSA: obstructive sleep apnea
PlGF: placental growth factor
POSA: pregnancy-associated obstructive sleep apnea
PT: positional therapy
RDI: respiratory disturbance index
REDCap: Research Electronic Data Capture
RR: relative risk
SDB: sleep-disordered breathing
sFlt-1: soluble fms-like tyrosine Kinase 1
SOMANZ: Society of Obstetric Medicine of Australia and New Zealand
SPIRIT: Standard Protocol Items: Recommendations for Interventional Trials
STARD: Standards for Reporting of Diagnostic Accuracy
STOP-BANG: snoring, tiredness, observed apnea, high BP (STOP) and snoring, tiredness, observed apnea, high BP-BMI, age, neck circumference, and gender
SWSLHD HREC: South Western Sydney Local Health District Human Research and Ethics Committee
TNFα: tumor necrosis factor alpha

## Data Availability

Data generated during this trial will be available following the completion and publication of the trial from the first and last authors upon reasonable request and approval by the local ethics committee*.*
