# Supplementary material for: Effect of Continuous Positive Airway Pressure or Positional Therapy Compared to Control for Treatment of Obstructive Sleep Apnea on the Development of Gestational Diabetes Mellitus in Pregnancy: Protocol for Feasibility Randomized Controlled Trial
Source: JMIR Res Protoc. 2025 Apr 11;14:e51434. doi: 10.2196/51434 (PMC12032501; doi:10.2196/51434)
Supplement: Multimedia Appendix 1 [file resprot_v14i1e51434_app1.pdf]

A Screening Eligibility Questionnaire Preconsent

Record ID	______________________
Today's Date (to be completed by investigator)	______________________
Investigator Name	______________________

We are conducting a research study on whether snoring and sleep apnoea affects the risk of diabetes during pregnancy.
This is a screening questionnaire to confirm whether or not you are eligible to take part in our study.
Please answer the following questions.

What is your date of birth?	______________________
Calculated Age	______________________<br>(Calculated value - do not enter data)
When is your expected date of delivery?<br>(Either from last menstrual period or from ultrasound)	______________________

How far along are you in your current pregnancy? (from LMP)

Gestation (weeks)	______________________
Gestation (days)	______________________
Are you having twins, triplets or more?	◯ Yes<br>◯ No
Do you know if your baby has Down's syndrome or another chromosomal abnormality?	◯ Yes<br>◯ No

We will now be asking some questions about your general background health.

Do you have confirmed gestational diabetes?<br>(i.e. Do you already have diabetes now?)	◯ Yes<br>◯ No<br>(If you have Type 1 diabetes you should answer 'no'.)
Have you had gestational diabetes (diabetes during pregnancy) in the past?	◯ Yes<br>◯ No

Do you have Type 1 or Type 2 diabetes?	◯ Yes<br>◯ No
Do you have a family history of diabetes?<br>(1st degree relative with diabetes or a sister with gestational diabetes)	◯ Yes<br>◯ No
Do you currently have confirmed preeclampsia?	◯ Yes<br>◯ No
Have you or your Mum or your sister ever had preeclampsia?	◯ Yes<br>◯ No
Do you have any kidney problems?	◯ Yes<br>◯ No
Has a doctor or other health professional ever told that you have sleep apnoea?	◯ Yes<br>◯ No
Are you currently on any treatment for sleep apnoea?	◯ Yes<br>◯ No
Do you snore at least three times a week?	◯ Yes<br>◯ No
Have anyone observed you stop breathing or choking/gasping during your sleep?	◯ Yes<br>◯ No

We will now measure your height and weight.

Height (cm)	______________________
Weight (kg)	______________________
Calculated BMI	______________________<br>(Calculated value - do not enter data)

Screening (Eligibility) Questionnaire - OUTCOME

You are eligible to take part in our study, "Diagnosis and management of sleep disordered breathing in pregnancy". Please review the Participant Information Sheet to help decide whether or not you would like to continue.

Has consent been obtained to continue?	◯ Yes<br>◯ No - Declined further participation<br>◯ No - Other (please add comment)
Comments	______________________

You have completed all questions required.
*** You are not eligible to take part in the rest of this study ***
Thank you for taking part in this survey.
