# Supplementary material for: Effect of Continuous Positive Airway Pressure or Positional Therapy Compared to Control for Treatment of Obstructive Sleep Apnea on the Development of Gestational Diabetes Mellitus in Pregnancy: Protocol for Feasibility Randomized Controlled Trial
Source: JMIR Res Protoc. 2025 Apr 11;14:e51434. doi: 10.2196/51434 (PMC12032501; doi:10.2196/51434)
Supplement: Multimedia Appendix 2 [file resprot_v14i1e51434_app2.pdf]

B Baseline Information Postconsent

Record ID ______________________

Participant Initials ______________________

Gravidity (G?) ______________________

Parity (P?) ______________________

Baseline measurements

Systolic Blood Pressure (mmHg) ______________________

Diastolic Blood Pressure (mmHg) ______________________

Neck circumference (cm) ______________________

Mallampati class
- ◯ Class 1
- ◯ Class 2
- ◯ Class 3
- ◯ Class 4

Baseline urinalysis (dipstick) - Protein
- ◯ Negative
- ◯ Trace
- ◯ 30 mg/dL (+)
- ◯ 100 mg/dL (++)
- ◯ 300 mg/dL (+++)
- ◯ >2000 mg/dL (++++)

Sleep-related Questions

Do you snore loudly (loud enough to be heard through closed doors or your bed-partner elbows you for snoring at night?)
- ◯ Yes
- ◯ No

Do you feel tired, fatigued or sleepy during the daytime (such as falling asleep driving or talking to someone)?
- ◯ Yes
- ◯ No

Has anyone observed you stop breathing or choking/gasping during your sleep? *HIDDEN* ______________________

Do you have or are you being treated for high blood pressure?
- ◯ Yes
- ◯ No

BMI more than 35 kg/m2? *HIDDEN* ________________________________

Age over 50 years old? *HIDDEN* ________________________________

Neck circumference greater than 40 cm? *HIDDEN* ________________________________

Calculated STOP-BANG total score ________________________________
(Calculated field - do not enter data)

Age (Facco) *HIDDEN* ________________________________

BMI (Facco) *HIDDEN* ________________________________

Chronic hypertension (Facco) *HIDDEN* ________________________________

Frequent snoring (Facco) *HIDDEN* ________________________________

Calculated pregnancy-specific screening tool score ________________________________
(Calculated field - do not enter data)

EPWORTH SLEEPINESS SCALE
How likely are you to doze off or fall asleep in the following situations, in contrast to feeling just tired? This refers to your usual way of life in recent times. Even if you haven't done some of these things recently, try to work out how they would have affected you. Use the following scale to choose the most appropriate option for each situation:
0 = would NEVER doze
1 = SLIGHT CHANCE of dozing
2 = MODERATE CHANCE of dozing
3 = HIGH chance of dozing
It is important that you answer each question as best as you can.

	0 = NEVER	1 = SLIGHT	2 = MODERATE	3 = HIGH
Sitting and reading	○	○	○	○
Watching TV	○	○	○	○
Sitting, inactive in a public place (e.g. a theatre or a meeting)	○	○	○	○

As a passenger in a car for an hour without a break	◯	◯	◯	◯
Lying down to rest in the afternoon when circumstances permit	◯	◯	◯	◯
Sitting and talking to someone	◯	◯	◯	◯
Sitting quietly after a lunch without alcohol	◯	◯	◯	◯
In a car, while stopped for a few minutes in traffic	◯	◯	◯	◯

Calculated ESS score (total)

____
(Calculated field - do not enter data)

Additional baseline information

Has your mum or sister ever had diabetes or hyperglycaemia during pregnancy?
◯ Yes
◯ No

Have you ever had a previous pregnancy where your baby weighed 4.5 kg or more at birth?
◯ Yes
◯ No

Do you have polycystic ovarian syndrome (PCOS)?
◯ Yes
◯ No

Are you currently taking oral (tablet) steroid therapy regularly for any reason? This does not include inhalers/steroid creams.
◯ Yes
◯ No

What medications do you take regularly? Please include all prescription medicines, over-the-counter medicines including vitamins and supplements, inhalers/nebulised medicines, creams/topical patches and medicines requiring injection.
____

How would you describe your ethnic background?
◯ Caucausian
◯ Aboriginal/Torres Strait Islander
◯ Asian
◯ Indian/Subcontinental
◯ Middle Eastern
◯ African (non-white)
◯ Other
◯ Polynesian
