# Supplementary material for: Effect of Continuous Positive Airway Pressure or Positional Therapy Compared to Control for Treatment of Obstructive Sleep Apnea on the Development of Gestational Diabetes Mellitus in Pregnancy: Protocol for Feasibility Randomized Controlled Trial
Source: JMIR Res Protoc. 2025 Apr 11;14:e51434. doi: 10.2196/51434 (PMC12032501; doi:10.2196/51434)
Supplement: Multimedia Appendix 6 [file resprot_v14i1e51434_app6.pdf]

PSG 28-32 weeks gestation questionnaire

Please complete the survey below related to the laboratory sleep test

Thank you!

Did you complete the Hospital Laboratory Sleep Study?

- ◯ Yes
- ◯ No

If No, please provide reason (optional_

____

Overall, how was the Hospital Laboratory Sleep Study? (Ease of use)

- ◯ Very difficult to use
- ◯ Difficult to use
- ◯ Neutral
- ◯ Easy to use
- ◯ Very easy to use

Why? (optional)

____

Overall, how was the Hospital Laboratory Sleep Study? (Convenience)

- ◯ Very inconvenient
- ◯ Inconvenient
- ◯ Neutral
- ◯ Convenient
- ◯ Very Convenient

Comments? (optional)

____

If required, how acceptable would you find repeating this test?

- ◯ Very unacceptable
- ◯ Unacceptable
- ◯ Neutral
- ◯ Acceptable
- ◯ Very acceptable

What were the main reasons for your answer? (optional)

____

Thankyou for completing this questionnaire!
