# Supplementary material for: Effect of Continuous Positive Airway Pressure or Positional Therapy Compared to Control for Treatment of Obstructive Sleep Apnea on the Development of Gestational Diabetes Mellitus in Pregnancy: Protocol for Feasibility Randomized Controlled Trial
Source: JMIR Res Protoc. 2025 Apr 11;14:e51434. doi: 10.2196/51434 (PMC12032501; doi:10.2196/51434)
Supplement: Multimedia Appendix 7 [file resprot_v14i1e51434_app7.pdf]

Somte 28-32 weeks gestation questionnaire

Please complete the survey below regarding the SOMTE home sleep test

Thank you!

Did you complete the Somte home polysomnopgraphy?	◯ Yes<br>◯ No
If No, please provide reason (optional_	__________________________________
Did you have difficulty completing the SOMTE home polysomnography?	◯ Yes<br>◯ No
If Yes, please describe the difficulty (optional)	__________________________________
Did you need any phone support during the SOMTE Home polysomnography?	◯ Yes<br>◯ No
If you used phone support, was the phone support helpful?	◯ Very Unhelpful<br>◯ Unhelpful<br>◯ Neutral<br>◯ Helpful<br>◯ Very Helpful
Comments? (Optional)	__________________________________
Overall, how was the SOMTE home polysomnography? (Ease of use)	◯ Very difficult to use<br>◯ Difficult to use<br>◯ Neutral<br>◯ Easy to use<br>◯ Very easy to use
Why? (optional)	__________________________________
Overall, how was the SOMTE home polysomnography? (Convenience)	◯ Very inconvenient<br>◯ Inconvenient<br>◯ Neutral<br>◯ Convenient<br>◯ Very Convenient
Comments? (optional)	__________________________________

If required, how acceptable would you find repeating this test?	◯ Very unacceptable<br>◯ Unacceptable<br>◯ Neutral<br>◯ Acceptable<br>◯ Very acceptable
What were the main reasons for your answer? (optional)	______________________________________

Thankyou for completing this questionnaire!
