# Supplementary material for: Effect of Continuous Positive Airway Pressure or Positional Therapy Compared to Control for Treatment of Obstructive Sleep Apnea on the Development of Gestational Diabetes Mellitus in Pregnancy: Protocol for Feasibility Randomized Controlled Trial
Source: JMIR Res Protoc. 2025 Apr 11;14:e51434. doi: 10.2196/51434 (PMC12032501; doi:10.2196/51434)
Supplement: Multimedia Appendix 8 [file resprot_v14i1e51434_app8.pdf]

Apnealink 28-32 weeks gestation questionnaire

Please complete the survey below regarding the apnealink home sleep test

Thank you!

Did you complete the apnealink home test?
- ◯ Yes
- ◯ No

If No, please provide reason (optional)

____

Did you have difficulty completing the apnealink Home Sleep test?
- ◯ Yes
- ◯ No

If Yes, please describe the difficulty (optional)

____

Did you need any phone support during the apnealink Home Sleep test?
- ◯ Yes
- ◯ No

If you used phone support, was the phone support helpful?
- ◯ Very Unhelpful
- ◯ Unhelpful
- ◯ Neutral
- ◯ Helpful
- ◯ Very Helpful

Comments? (Optional)

____

Overall, how was the Apnealink home sleep test? (Ease of use)
- ◯ Very difficult to use
- ◯ Difficult to use
- ◯ Neutral
- ◯ Easy to use
- ◯ Very easy to use

Why? (optional)

____

Overall, how was the Apnealink home Sleep test? (Convenience)	◯ Very inconvenient<br>◯ Inconvenient<br>◯ Neutral<br>◯ Convenient<br>◯ Very Convenient
Comments? (optional)	______________________________________
If required, how acceptable would you find repeating this test?	◯ Very unacceptable<br>◯ Unacceptable<br>◯ Neutral<br>◯ Acceptable<br>◯ Very acceptable
What were the main reasons for your answer? (optional)	______________________________________

Thankyou for completing this questionnaire!
