# Supplementary material for: Effect of Continuous Positive Airway Pressure or Positional Therapy Compared to Control for Treatment of Obstructive Sleep Apnea on the Development of Gestational Diabetes Mellitus in Pregnancy: Protocol for Feasibility Randomized Controlled Trial
Source: JMIR Res Protoc. 2025 Apr 11;14:e51434. doi: 10.2196/51434 (PMC12032501; doi:10.2196/51434)
Supplement: Multimedia Appendix 9 [file resprot_v14i1e51434_app9.pdf]

Preferred test questionnaire

Please complete the survey below

Thank you!

	Please rank the three tests in order of preference 1=most preferred, 3= least preferred			
		1	2	3
1)	Hospital laboratory sleep study	◯	◯	◯
2)	Home sleep study SOMTE	◯	◯	◯
3)	Home sleep study Apnealink	◯	◯	◯
