# Supplementary material for: Effect of Continuous Positive Airway Pressure or Positional Therapy Compared to Control for Treatment of Obstructive Sleep Apnea on the Development of Gestational Diabetes Mellitus in Pregnancy: Protocol for Feasibility Randomized Controlled Trial
Source: JMIR Res Protoc. 2025 Apr 11;14:e51434. doi: 10.2196/51434 (PMC12032501; doi:10.2196/51434)
Supplement: Multimedia Appendix 10 [file resprot_v14i1e51434_app10.pdf]

CPAP therapy Q 16-20 weeks gestation

Please complete the survey below.

Thank you!

Were the CPAP set-up instructions clear?	◯ Very unclear<br>◯ Unclear<br>◯ Neutral<br>◯ Clear<br>◯ Very clear
Comments (optional)	__________________________________
Did you need any phone support to use CPAP during the past 7 days?	◯ Yes<br>◯ No
If you used phone support, was the phone support helpful?	◯ Very Unhelpful<br>◯ Unhelpful<br>◯ Neutral<br>◯ Helpful<br>◯ Very Helpful
Comments? (Optional)	__________________________________
Have you encountered difficulty using CPAP in the past 7 days?	◯ Yes<br>◯ No
If you encountered difficulty using CPAP therapy in the past 7 days, what were the main reasons? (tick as many as you feel apply to you)	☐ Claustrophobia<br>☐ Anxiety<br>☐ Nasal congestion/blockage<br>☐ Mask Leak<br>☐ Mask problems<br>☐ Family commitments<br>☐ work commitments<br>☐ pregnancy related issues<br>☐ difficulty using CPAP therapy<br>☐ lack of support by spouse/partner<br>☐ Children in the home<br>☐ Other reason
If children in the home caused difficulty to use CPAP therapy, please specify the number of children in the home	◯ 1<br>◯ 2<br>◯ 3<br>◯ 4<br>◯ 5<br>◯ 6<br>◯ 7<br>◯ 8<br>◯ 9<br>◯ 10

If children in the home caused difficulty using CPAP therapy, please specify the ages of the children	☐ 0<br>☐ 1<br>☐ 2<br>☐ 3<br>☐ 4<br>☐ 5<br>☐ 6<br>☐ 7<br>☐ 8<br>☐ 9<br>☐ 10<br>☐ 11<br>☐ 12<br>☐ 13<br>☐ 14<br>☐ 15<br>☐ 16<br>☐ 17<br>☐ 18<br>☐ over 18
if other, please specify (optional)	__________________________________
If you needed CPAP therapy in a future pregnancy, how likely would you be to use it again?	◯ Very unlikely<br>◯ Unlikely<br>◯ Neutral<br>◯ Likely<br>◯ Very likely
Overall, how acceptable did you find CPAP therapy?	◯ Very unacceptable<br>◯ Unacceptable<br>◯ Neutral<br>◯ Acceptable<br>◯ Very acceptable

Thankyou for completing this questionnaire!
