# Supplementary material for: Effect of Continuous Positive Airway Pressure or Positional Therapy Compared to Control for Treatment of Obstructive Sleep Apnea on the Development of Gestational Diabetes Mellitus in Pregnancy: Protocol for Feasibility Randomized Controlled Trial
Source: JMIR Res Protoc. 2025 Apr 11;14:e51434. doi: 10.2196/51434 (PMC12032501; doi:10.2196/51434)
Supplement: Multimedia Appendix 12 [file resprot_v14i1e51434_app12.pdf]

position therapy Q 36 weeks gestation

Please complete the survey below.

Thank you!

Were the set-up instructions clear?

- ◯ Very unclear
- ◯ Unclear
- ◯ Neutral
- ◯ Clear
- ◯ Very clear

Comments (optional)

____

Did you need any phone support to use position therapy during the past 7 days?

- ◯ Yes
- ◯ No

If you used phone support, was the phone support helpful?

- ◯ Very Unhelpful
- ◯ Unhelpful
- ◯ Neutral
- ◯ Helpful
- ◯ Very Helpful

Comments? (Optional)

____

Have you encountered difficulty using positional therapy in the past 7 days?

- ◯ Yes
- ◯ No

If you encountered difficulty using position therapy in the past 7 days, what were the main reasons? (tick as many as you feel apply to you)

- ☐ Claustrophobia
- ☐ Discomfort
- ☐ Anxiety
- ☐ Family commitments
- ☐ Work commitments
- ☐ Pregnancy related issues
- ☐ Difficulty using position therapy
- ☐ Lack of support by spouse / partner
- ☐ Children in the home
- ☐ Other reason

If children in the home caused difficulty to use position therapy therapy, please specify the number of children in the home

- ◯ 1
- ◯ 2
- ◯ 3
- ◯ 4
- ◯ 5
- ◯ 6
- ◯ 7
- ◯ 8
- ◯ 9
- ◯ 10

If children in the home caused difficulty using positional therapy, please specify the ages of the children	☐ 0<br>☐ 1<br>☐ 2<br>☐ 3<br>☐ 4<br>☐ 5<br>☐ 6<br>☐ 7<br>☐ 8<br>☐ 9<br>☐ 10<br>☐ 11<br>☐ 12<br>☐ 13<br>☐ 14<br>☐ 15<br>☐ 16<br>☐ 17<br>☐ 18<br>☐ over 18
if other, please specify (optional)	________________________________
If you needed position therapy in a future pregnancy, how likely would you be to use it again?	◯ Very unlikely<br>◯ Unlikely<br>◯ Neutral<br>◯ Likely<br>◯ Very likely
Overall, how acceptable did you find position therapy?	◯ Very unacceptable<br>◯ Unacceptable<br>◯ Neutral<br>◯ Acceptable<br>◯ Very acceptable

Thankyou for completing this questionnaire!
