# Supplementary material for: Effect of Continuous Positive Airway Pressure or Positional Therapy Compared to Control for Treatment of Obstructive Sleep Apnea on the Development of Gestational Diabetes Mellitus in Pregnancy: Protocol for Feasibility Randomized Controlled Trial
Source: JMIR Res Protoc. 2025 Apr 11;14:e51434. doi: 10.2196/51434 (PMC12032501; doi:10.2196/51434)
Supplement: Multimedia Appendix 13 [file resprot_v14i1e51434_app13.pdf]

Position therapy Q 16-20 weeks gestation

Please complete the survey below.

Thank you!

Question	Response
Were the set-up instructions clear?	◯ Very unclear<br>◯ Unclear<br>◯ Neutral<br>◯ Clear<br>◯ Very clear
Comments (optional)	__________________________________
Did you need any phone support to use position therapy during the past 7 days?	◯ Yes<br>◯ No
If you used phone support, was the phone support helpful?	◯ Very Unhelpful<br>◯ Unhelpful<br>◯ Neutral<br>◯ Helpful<br>◯ Very Helpful
Comments? (Optional)	__________________________________
Have you encountered difficulty using positional therapy in the past 7 days?	◯ Yes<br>◯ No
If you encountered difficulty using position therapy in the past 7 days, what were the main reasons? (tick as many as you feel apply to you)	☐ Claustrophobia<br>☐ Discomfort<br>☐ Anxiety<br>☐ Family commitments<br>☐ Work commitments<br>☐ Pregnancy related issues<br>☐ Difficulty using position therapy<br>☐ Lack of support by spouse / partner<br>☐ Children in the home<br>☐ Other reason
If children in the home caused difficulty to use position therapy therapy, please specify the number of children in the home	◯ 1<br>◯ 2<br>◯ 3<br>◯ 4<br>◯ 5<br>◯ 6<br>◯ 7<br>◯ 8<br>◯ 9<br>◯ 10

If children in the home caused difficulty using positional therapy, please specify the ages of the children	☐ 0<br>☐ 1<br>☐ 2<br>☐ 3<br>☐ 4<br>☐ 5<br>☐ 6<br>☐ 7<br>☐ 8<br>☐ 9<br>☐ 10<br>☐ 11<br>☐ 12<br>☐ 13<br>☐ 14<br>☐ 15<br>☐ 16<br>☐ 17<br>☐ 18<br>☐ over 18
if other, please specify (optional)	____________________________________
If you needed position therapy in a future pregnancy, how likely would you be to use it again?	○ Very unlikely<br>○ Unlikely<br>○ Neutral<br>○ Likely<br>○ Very likely
Overall, how acceptable did you find position therapy?	○ Very unacceptable<br>○ Unacceptable<br>○ Neutral<br>○ Acceptable<br>○ Very acceptable

Thankyou for completing this questionnaire!
