# Supplementary material for: Effect of Continuous Positive Airway Pressure or Positional Therapy Compared to Control for Treatment of Obstructive Sleep Apnea on the Development of Gestational Diabetes Mellitus in Pregnancy: Protocol for Feasibility Randomized Controlled Trial
Source: JMIR Res Protoc. 2025 Apr 11;14:e51434. doi: 10.2196/51434 (PMC12032501; doi:10.2196/51434)
Supplement: Multimedia Appendix 5 [file resprot_v14i1e51434_app5.pdf]

		Pre-RCT up to 14 wks Gestation	Pregnancy and Obstructive Sleep Apnoea (POSA) Randomised Controlled Trial											
			RCT Study visits											
			Consent and Randomisation 12-16 wks gestation	Virtual Visit CPAP group only 3-7 days after commencing therapy	16-20 Wks Gestation	20-24 wks Gestation	24-28 Wks Gestation	28 Wks Gestation	28-32 Wks Gestation	32 Wks Gestation	34 Wks Gestation	36 Wks Gestation	Delivery	6-12 months post-partum
Procedures and data collection items	**Consent**	X	X											
	**Screening Eligibility Questionnaire Preconsent**	X												
	**Baseline Information Postconsent**	X												
	**Randomisation**		X											
	**Blood Tests**													
	Glucose Tolerance Test (GTT) (primary outcome)							X						
	Study bloods		X		X			X						X
	Biomarkers (secondary outcome)		X		X			X						X
	HbA1c for those diagnosed with diabetes (secondary outcome)							X				X		
	**Weight**													
	Maternal weight (secondary outcome)	X	X		X	X	X	X	X	X	X	X		
	Birthweight (secondary outcome)												X	
	Placental weight (secondary outcome)												X	
	**Urinalysis**	X	X		X	X	X	X	X	X	X	X		
	**Blood Pressure**	X	X		X	X	X	X	X	X	X	X	X	X
	**Ultrasounds**													
	Uterine Artery Blood Flow (Pulsatility Index)(secondary outcome)		X		X							X		
	Morphology				X									
	Growth Percentile (secondary outcome)											X		
	**Maternal Outcomes (Secondary outcome)**												X	X
	**Foetal Outcomes (Secondary outcome)**												X	X
	**Sleep Study Test**													
	Attended Polysomnography (CPAP or position therapy in situ for treatment group participants at 28 week PSG)	X							X					X
	Unattended Polysomnography	X							X					
	Home Limited Channel sleep study	X							X					
	**Download of treatment device**													
	Download of CPAP device			X	X	X	X	X	X	X	X	X	X	
	Download of position therapy device				X	X	X	X	X	X	X	X	X	
	**Questionnaires**													
	Attended Polysomnography Questionnaire	X							X					
	Home Polysomnography Questionnaire	X							X					
	Limited Channel Home sleep study Questionnaire	X							X					
	Preferred sleep test questionnaire	X							X					
	FOSQ-10		X		X							X		
	CPAP therapy device questionnaire				X							X		
	Position therapy device questionnaire				X							X		
